# Before Linnaeus and Darwin: the hierarchical taxonomy of Li Shizhen’s *Bencao Gangmu*

**DOI:** 10.3389/fphar.2026.1867126

**Published:** 2026-07-21

**Authors:** Tung-Hu Tsai

**Affiliations:** 1 School of Chinese Medicine, College of Medicine, National Yang Ming Chiao Tung University, Taipei, Taiwan; 2 School of Pharmacy, College of Pharmacy, Kaohsiung Medical University, Kaohsiung, Taiwan

**Keywords:** *Bencao Gangmu*, history of science, Li Shizhen, medical education, taxonomy, traditional Chinese medicine

## Abstract

**Background:**

The *Bencao Gangmu* (Compendium of Materia Medica, 1596), by Li Shizhen (1518–1593), is one of the most comprehensive pre-modern pharmacopoeias in the history of traditional medicine. Comprising 1,892 drug species organized into the inorganic, plant, and animal kingdoms, this foundational text of Chinese traditional pharmacology documents the therapeutic uses, morphological descriptions, and ecological contexts of medicinal substances—roughly 175–300 years before the formalized systems of Western taxonomy and natural history were established.

**Aim:**

This study examines the structural and epistemological parallels between Li Shizhen’s four-tiered classification system and the taxonomic frameworks of Carolus Linnaeus (1707–1778) and Charles Darwin (1809–1882), evaluates the historical transmission of the Compendium to Europe, and argues for integrating this text into modern medical and scientific education curricula.

**Methods:**

A comparative historical-textual analysis was conducted using the 1672 critical edition of the *Bencao Gangmu*, available through the National Central Library (Taiwan) and the Chinese Text Project digital archive (ctext.org), along with Open CourseWare materials from National Yang Ming Chiao Tung University. Li Shizhen’s four-tiered organizational hierarchy was evaluated against the Linnaean taxonomic system and the theory of evolution by natural selection.

**Results:**

Li Shizhen’s classification system (kingdom → department → category → species) predates the Linnaean taxonomic hierarchy by about 175 years and reflects empirically and morphologically grounded groupings consistent with what is now recognized as alpha taxonomy. The Compendium’s sequential ordering of organisms, attention to intraspecific variation, empiricist critical methodology, and ecologically grounded groupings collectively exhibit structural and epistemological features analogous to—though not mechanistically anticipating—elements of Darwinian natural history. These parallels are structural and analogical; the Compendium does not articulate a theory of evolutionary transformation, and its sequential ordering reflects a traditional Chinese cosmological framework rather than evolutionary genealogy.

**Conclusion:**

The *Bencao Gangmu* is a significant contribution to the global history of systematic natural inquiry, and its classificatory achievements merit scholarly recognition alongside those of Linnaeus and Darwin. Incorporating it into medical and scientific education curricula would deepen students’ understanding of the diverse cultural foundations of pharmacology, taxonomy, and evidence-based medical reasoning.

## 1 Introduction

The history of biological classification is often told as an exclusively European enterprise, tracing a linear progression from Aristotle’s animal groupings to the eighteenth-century Linnaean revolution. Yet this Eurocentric narrative obscures a parallel—and in many respects, anticipatory—tradition of systematic natural inquiry in China. It is important to acknowledge at the outset that this parallel tradition operated within a fundamentally different intellectual framework: sixteenth-century Chinese pharmacological scholarship was shaped by Confucian scholarly values, Daoist cosmology, and the clinical imperatives of the physician, while Enlightenment European taxonomy was driven by universalist ambitions, natural theology, and emerging experimental science. The comparison made in this paper is therefore between two distinct intellectual achievements emerging from incommensurable epistemological traditions, not between two versions of the same scientific program. Completed by the physician-naturalist Li Shizhen in 1578 and published posthumously in 1596, the *Bencao Gangmu* classifies 1,892 medicinal substances according to an explicit hierarchical logic that prefigures the binomial and polytomous systems later formalized in the West (Li, 1596).

Born into a family of physicians in Qizhou (present-day Hubei Province), Li Shizhen (1518–1593) devoted 27 years to field research, clinical observation, and scholarly synthesis. The resulting Compendium is a monumental text of approximately two million characters, spanning fifty-two volumes. It organizes 1,892 drug species into sixteen departments and sixty categories, accompanied by 11,096 prescriptions and 1,109 illustrations (Li, 1596; Needham and Lu, 2000). The work assimilated and critically revised more than forty earlier materia medica texts, dating back to the *Shennong Bencao Jing* (Classic of Materia Medica, c. 200 CE), while integrating insights from hundreds of historical, literary, and philosophical sources (Tang, 1989).

In the West, modern taxonomy is anchored in the work of Carolus Linnaeus (1707–1778), who formalized binomial nomenclature in Species Plantarum (1753) and Systema Naturae (10th edition, 1758). His hierarchical ranks—Kingdom, Phylum, Class, Order, Family, Genus, and Species—established a universal language for describing biodiversity that remains foundational to this day (Linnaeus, 1753). Charles Darwin (1809–1882) subsequently provided biological classification with its modern theoretical framework. In On the Origin of Species (1859), Darwin proposed natural selection and common ancestry, arguing that any satisfactory classification must ultimately reflect evolutionary genealogy (Darwin, 1859).

The chronological relationship among these figures positions Li Shizhen as a distant historical predecessor, preceding Linnaeus by roughly 175 years and Darwin by nearly three centuries. Although Darwin has been anecdotally reported to have described the Compendium as “the ancient Chinese encyclopedia” (China Medical University, 2026), this attribution remains unverified.

**FIGURE 1:**
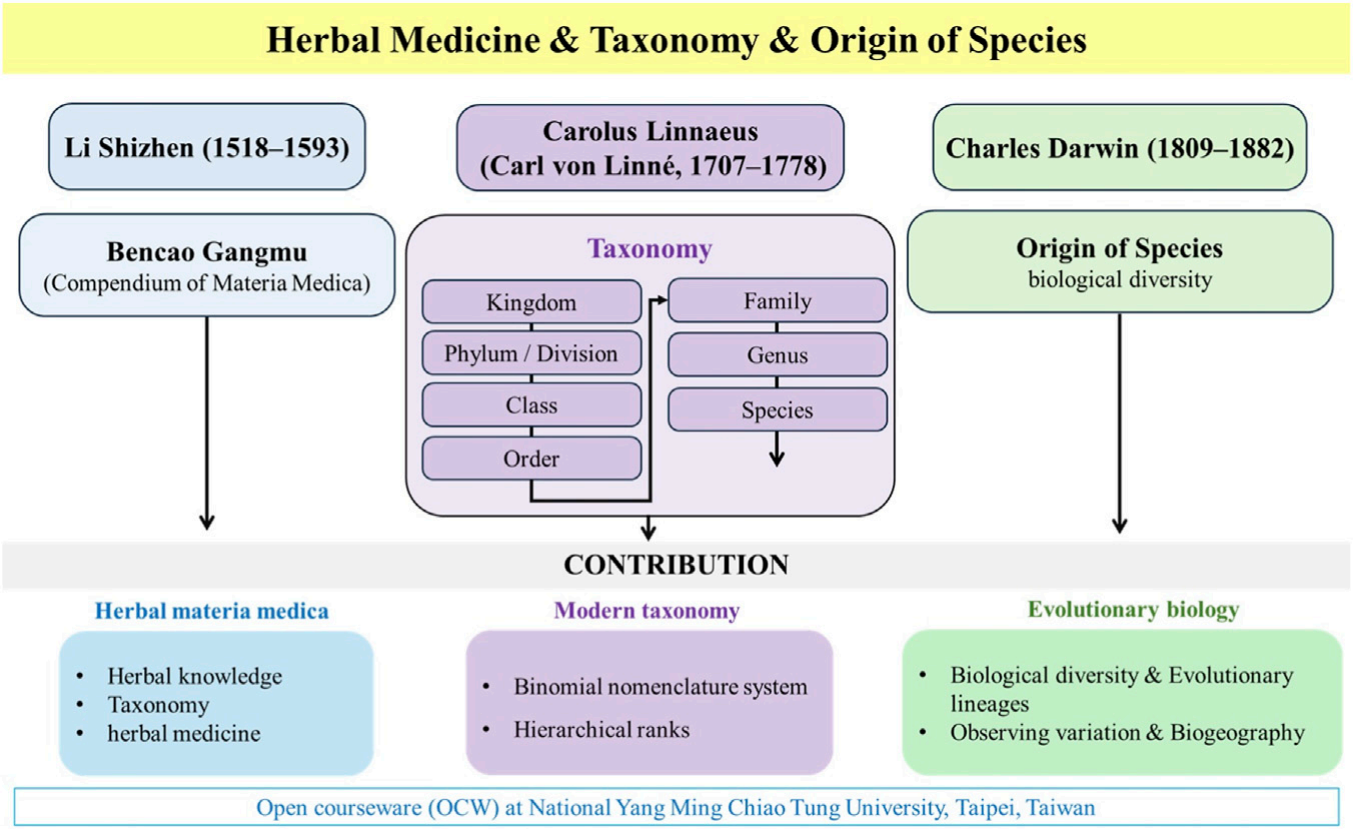
Presents a comparative timeline situating the major contributions of Li Shizhen, Linnaeus, and Darwin within the global history of natural inquiry.

Inscribed on the United Nations Educational, Scientific and Cultural Organization (UNESCO) Memory of the World Register in 2011, the Compendium is formally recognized as a document of global heritage (UNESCO, 2011). Despite this recognition, its contributions to the history of taxonomy and evolutionary thought remain markedly underrepresented in Western medical and scientific education. To address this gap, this paper analyzes the Compendium’s classification system in relation to Linnaean taxonomy and Darwinian evolutionary principles, evaluates evidence of its intellectual influence on European natural history, and argues for the pedagogical value of integrating this text into modern scientific curricula. We acknowledge at the outset, in response to peer review, that this paper functions as a critical historical review and interpretive scholarly analysis rather than a report of new empirical findings. All comparisons drawn between Li Shizhen’s pharmacopoeial system and the frameworks of Linnaeus and Darwin are structural and analogical in nature; they are offered as reasoned scholarly interpretation, not as empirical demonstration. Speculative arguments are explicitly flagged as such throughout, and the article should be read accordingly (Figure 1).

## 2 Materials and methods

This study employs a comparative historical-textual methodology. The primary source is the *Bencao Gangmu*, using the National Central Library’s authoritative 1672 edition, accessed via the Chinese Text Project digital archive and Tang Mingbang’s critical scholarly edition (Tang, 1989). The Open CourseWare (OCW) materials developed at National Yang Ming Chiao Tung University (NYCU), Taiwan, offer a unique vehicle for medical teaching through classical pharmacopeial scholarship. The analytical framework evaluates Li Shizhen’s four-tiered organizational logic—kingdom, department, category, and individual drug species—against two principal reference standards: the Linnaean taxonomic hierarchy, as codified in *Species Plantarum* (1753) and *Systema Naturae* (10th ed., 1758) (Linnaeus, 1753), and the theory of evolution by natural selection, as expounded in *On the Origin of Species* (1859) (Darwin, 1859).

Secondary literature was identified through a systematic search of PubMed, Web of Science, and Google Scholar. Search terms included '*Bencao Gangmu*’, 'Li Shizhen’, 'traditional Chinese medicine taxonomy’, 'history of classification’, and 'history of biology’, with no date restrictions. In addition, key reference works on the history of Chinese science (Needham and Lu, 2000), the history of taxonomy (Stace, 1989; de Candolle, 1813), the philosophy of Chinese medicine (Unschuld, 1985), and medical education (Bleakley, 2015; Trullàs et al., 2022) were systematically reviewed. The educational implications of the Compendium were then assessed using the frameworks of evidence-based medical education and the One Health paradigm (Zinsstag et al., 2011). Because this research relied exclusively on historical textual analysis and involved no human participants, ethics approval was not required.

To enhance methodological transparency in response to peer review, we specify the following: (a) Textual passages were selected on the basis of three explicit criteria: (i) they directly describe Li Shizhen’s organizational rationale in his own words or in Tang (1989)’s scholarly gloss; (ii) they contain classifying or grouping language that can be operationally compared to Linnaean or Darwinian categories; or (iii) they have been previously cited in the secondary sinological literature (Needham and Lu, 2000; Unschuld, 1985) as methodologically significant. (b) “Structural parallel” is defined narrowly as the presence of analogous organizational logic—hierarchical nesting, morphological grouping, or empiricist error-correction—without implying causal connection or theoretical equivalence. (c) The analytical framework follows the comparative epistemology tradition in the history and philosophy of science, as practiced in Needham (1954–2015) and Unschuld (1985); no formal computational or quantitative methodology was applied, and the analysis is explicitly interpretive. (d) Interpretive bias was minimized by systematically including both parallels and divergences for each comparison, by grounding all claims in documented textual evidence rather than inference, and by subjecting all interpretive conclusions to the explicit caveat framework in Section 4.3.

## 3 Results

### 3.1 The compendium of materia medica: scope and scientific architecture

The title itself encodes Li Shizhen’s methodological program. The term *bencao* denotes the traditional Chinese pharmacological canon, while *gangmu* (literally 'cardinal principle and subsidiary details’) invokes the classical Chinese metaphor of a fishing net—the *gang* being the main rope that, when lifted, raises all the subsidiary mesh, the *mu*. Li Shizhen articulates this logic explicitly in the preface: “For each drug I establish a correct name as the *gang* (cardinal principle), and append alternative names as the *mu* (subsidiary details)” (Li, 1596). This recursive hierarchical structure—*gang* setting the organizing principle, and *mu* providing the elaboration—operates across four nested scales.

#### 3.1.1 Kingdoms (the broadest level)

Li Shizhen divides all medicinal substances into three primary kingdoms. The inorganic kingdom encompasses water, fire, earth, and minerals (four departments); the plant kingdom encompasses grasses, grains, vegetables, fruits, trees, and manufactured plant products (six departments); and the animal kingdom encompasses insects, scaled animals, shelled animals, birds, beasts, and humans (six departments). This tripartite division of sixteen departments broadly corresponds to the mineral, vegetable, and animal kingdoms that Linnaeus would later enshrine in *Systema Naturae* (Linnaeus, 1753; Li, 1596; Stafleu, 1971).

#### 3.1.2 Departments (organismal complexity)

Within each kingdom, departments provide the next level of organization. Their ordering is not arbitrary; Li Shizhen articulates an explicit rationale in which water and fire are placed first as “the progenitors of all things,” followed by earth as “the mother of all things,” and metals and stones emerging “from the earth.” Plant departments are arranged “from small to large,” while animal departments are ordered “from humble to noble,” culminating in the human department. This deliberate sequence encodes a proto-hierarchical view of organismal complexity (Tang, 1989; Li, 1596).

#### 3.1.3 Categories (morphological and ecological affinities)

The sixty categories further subdivide the departments into natural groupings based on morphological and ecological affinities. Within the grass department, for example, Li Shizhen distinguishes mountain, aromatic, lush, toxic, creeping, water, stone, and miscellaneous grasses, as well as mosses—categories that bear a recognizable relationship to the groupings employed by later botanists (Needham and Lu, 2000). Similarly, the animal departments divide scaled animals into four categories: dragons, snakes, fish, and scaleless fish. While occasionally blending mythological and empirical subjects, this grouping demonstrates systematic attention to morphological characteristics.

#### 3.1.4 Individual drugs (standardized descriptions)

Within each category, individual drugs are described using a rigorous, standardized eight-section format.

1. Shì-míng (etymological explanation of the name)
2. jì-jié (compilation of earlier descriptions with geographical distribution)
3. Xiū-zhì (collection seasons and processing methods)
4. Qí-wěi (four energies: cold, hot, warm, cool; and five flavors: pungent, sour, sweet, bitter, salty)
5. Zhǔ-zhì (therapeutic indications)
6. Fā-míng (elaboration of therapeutic mechanisms)
7. Zhèng-wù (correction of prior errors)
8. Fù-fāng (appended prescriptions)

**FIGURE 2:**
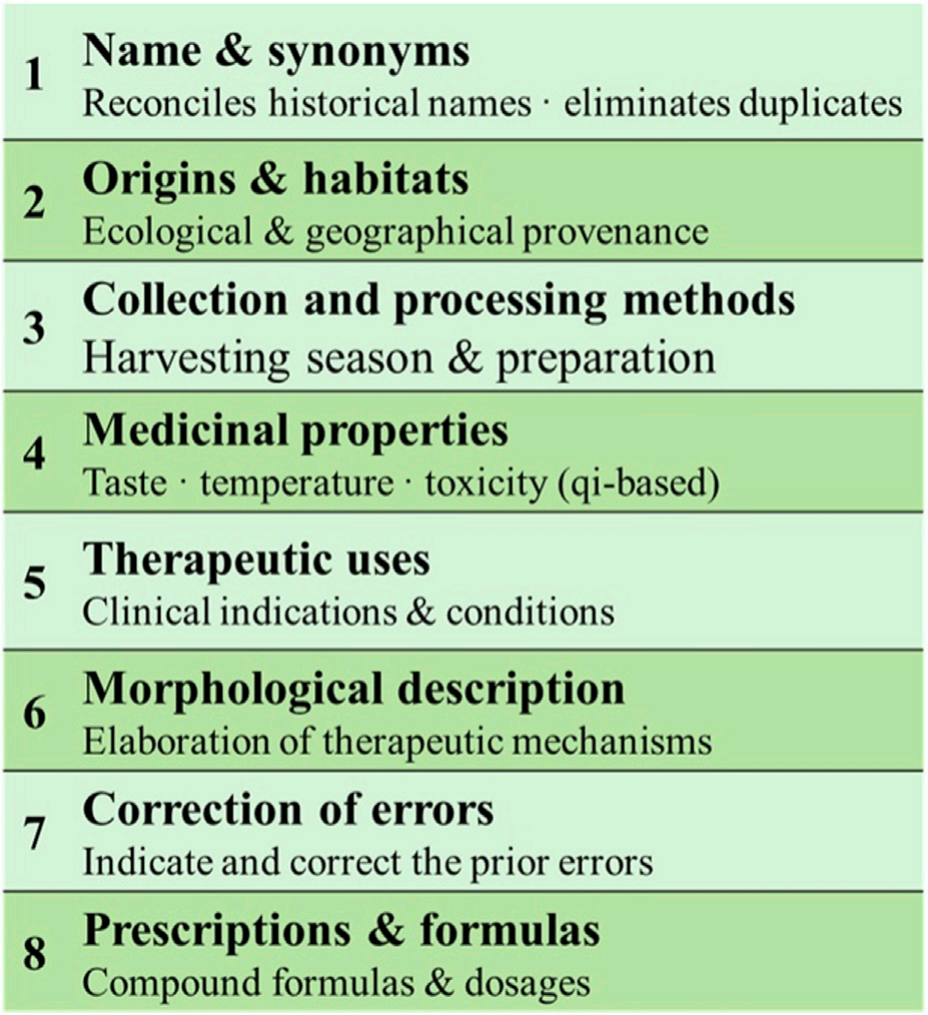
Illustrates the standardized eight-section format used to describe individual drugs within the *Bencao Gangmu*.

This meticulous format closely resembles the standardized taxonomic descriptions (protologues) used in modern botanical nomenclature, which require a diagnosis, synonymy, distribution, and type specimen information. It should be noted, however, that a formal protologue additionally requires a Latin diagnosis, a designated type specimen, and publication in a recognized botanical outlet—criteria that Li Shizhen’s format does not fulfill. The parallel is therefore functional and heuristic rather than formally equivalent (Li, 1596) (Figure 2).

### 3.2 Li Shizhen’s classification compared with linnaean taxonomy

**FIGURE 3:**
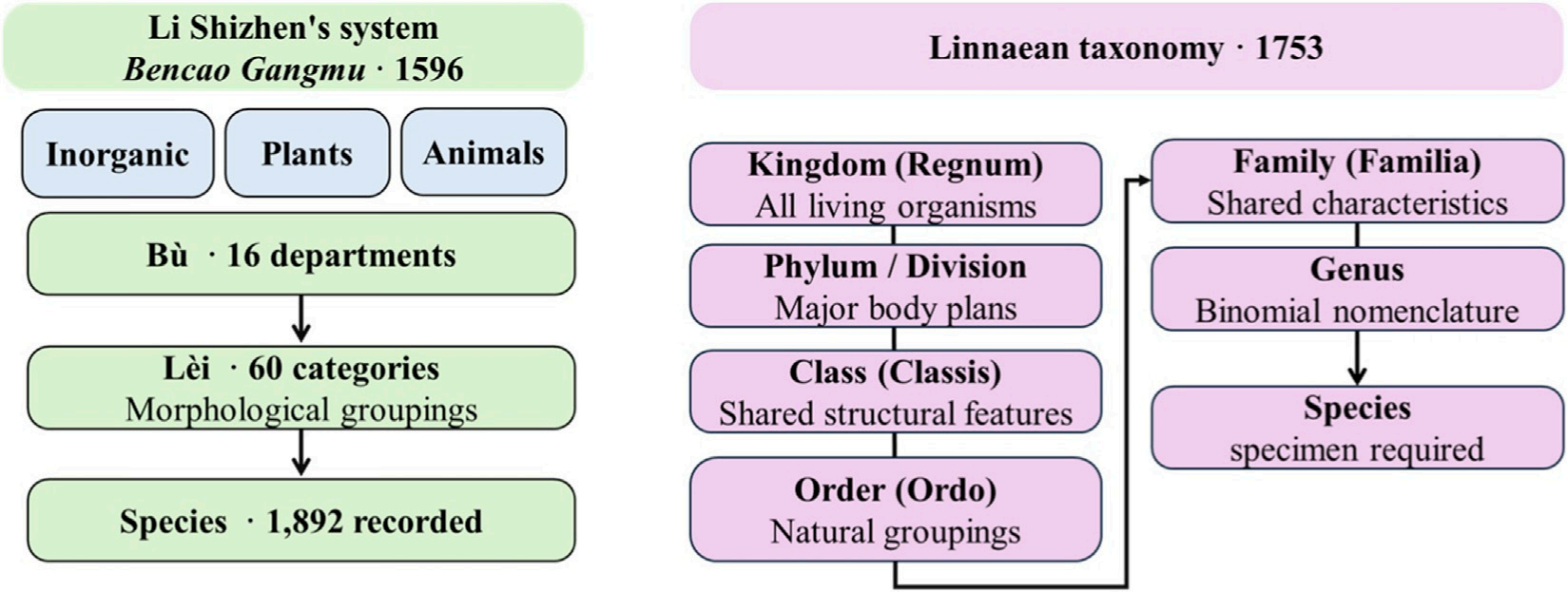
Provides a side-by-side structural comparison of Li Shizhen’s four-tiered classification hierarchy (kingdom → department → category → species) with the Linnaean taxonomic ranks, visually for the comparative analysis in Section 3.2.

The structural parallel between Li Shizhen’s four-tiered system (kingdom → department → category → species) and Linnaeus’s hierarchical ranks (kingdom → phylum/division → class → order → family → genus → species) is striking. The two systems are fundamentally different in purpose, scope, and philosophical foundation: the *Bencao Gangmu* is a pharmacopeial reference work whose primary goal is to facilitate a physician’s identification and retrieval of therapeutic substances within the Chinese medical tradition, whereas Linnaeus’s system is a universal biological taxonomy designed to reflect the natural relationships among all living organisms and to provide a stable nomenclatural framework for global scientific communication. Li Shizhen was not constructing a universal theory of biodiversity; his primary purpose was pharmacological—to equip physicians and apothecaries with a reliable reference work. Nevertheless, the classificatory logic he applied was empirical, hierarchical, and grounded in the observable properties of organisms and substances, meeting the fundamental requirements of what is now termed alpha taxonomy (Stace, 1989) (Figure 3).

Linnaeus himself emerged from a similar pharmacological and medical tradition. His early botanical work was motivated in part by the need to accurately identify medicinal plants, and Species Plantarum was explicitly intended to replace the unwieldy polynomial names used in pharmacy with concise two-word designations (Linnaeus, 1753). Thus, both Li Shizhen and Linnaeus approached taxonomy as a practical necessity before elevating it to a theoretical program. As elaborated in Section 3.2.1, however, this shared practical starting point should not be mistaken for a shared method or aim: their mature systems answered fundamentally different questions, and neither figure was “empiricist” in an unqualified sense. The primary divergence lies in Linnaeus’s explicit ambition to describe all of nature within a single universal system—shaped by Enlightenment universalism—whereas Li Shizhen’s system, though encyclopedic, remained firmly anchored in the Chinese medical tradition.

At the taxonomic level, these parallels are illuminating. Consider the medicinal plant *Scutellaria baicalensis* Georgi [Lamiaceae/huangqin], which appears in both the Compendium and modern botanical nomenclature. In the Compendium, it is placed in the grass department under the mountain grass category and described with precise attention to morphological characters such as leaf shape, stem color, and root structure—the very traits that define the genus *Scutellaria* in Linnaean taxonomy. The modern binomial *Scutellaria baicalensis* Georgi places the plant in the family Lamiaceae, genus *Scutellaria*, and species *baicalensis*, fully corroborating Li Shizhen’s grouping of yellow-rooted, labiate-flowered herbs (Zhao et al., 2016).

A critical difference between the two systems, however, lies in the principles of priority and nomenclatural stability. Linnaeus’s binomial system, codified in the International Code of Nomenclature for algae, fungi, and plants (ICN) and its zoological counterpart (ICZN), provides formal rules for resolving conflicts between competing names and for anchoring names to type specimens (Turland et al., 2018). Li Shizhen’s framework lacks such a formal apparatus; the names in the Compendium are Chinese vernacular or classical terms subject to regional and historical variation. Nevertheless, the Compendium’s Shì-míng sections—which systematically list synonyms, variant characters, and etymological derivations—serve a purpose analogous to the synonymy sections of modern taxonomic revisions by providing a comprehensive historical record of a name’s usage.

Another striking parallel concerns the concept of the “type specimen.” Modern taxonomy requires that each species name be anchored to a physical specimen deposited in a herbarium or museum, enabling future researchers to verify the organism’s identity. Although Li Shizhen did not formally deposit physical specimens, his descriptions in the jì-jié sections offer detailed accounts of morphology, phenology, and geographical provenance that often surpass the accuracy of contemporary European herbals. His willingness to challenge erroneous identifications, such as his critique of the misidentification of *Glycyrrhiza uralensis* Fisch. ex DC. [Fabaceae/gancao] in earlier texts reflects the same commitment to nomenclatural precision that drives modern taxonomic revision (Li, 1596; Tang, 1989).

Finally, the Compendium reflects what taxonomists now call a “natural grouping” rather than an “artificial grouping”. An artificial classification organizes organisms by a single, easily observed trait, regardless of broader similarities—a prime example is Linnaeus’s early sexual system, which grouped plants strictly by stamen count and arrangement. By contrast, a natural classification clusters organisms by overall similarity across multiple traits, reflecting their true morphological relationships. Li Shizhen’s categorization of plants by growth habit, ecology, and morphological affinity—such as grouping aromatic grasses together or separating aquatic from terrestrial herbs—closely aligns with this natural approach. His methodology anticipates the “natural systems” later championed by European botanists such as Antoine Laurent de Jussieu and Augustin Pyramus de Candolle, who similarly sought to reveal the inherent order of flora rather than impose an arbitrary framework upon it (de Candolle, 1813).

#### 3.2.1 Two organizing principles: a pragmatic-empiricist materia medica versus a universalist natural system

A structural resemblance between the two hierarchies should not obscure a deeper divergence in their organizing principles, which determine what each system contains, how its units are defined, and what would count as success. The *Bencao Gangmu* is organized by therapeutic use and by cosmological order: its primary unit is not the organism but the medicinal substance (*yao;* drug), and an entry earns its place by being pharmacologically or cosmologically relevant rather than by exemplifying the diversity of living forms. This is why the Compendium accommodates categories that have no counterpart in an organismal taxonomy—water, fire, earth, minerals, manufactured plant products, and human-derived substances—and orders its departments along an axis of cosmological priority (“progenitors of all things,” “mother of all things”) rather than of morphological affinity alone. It is, in this sense, a systematic inquiry into what is medically useful, and its classificatory criterion is retrievability for the physician.

Linnaeus’s system answers a different question. Its aim is universal: to impose a single, stable order on all living diversity and to provide a nomenclature adequate for global scientific communication. Its unit is the taxon, defined by similarity—in the sexual system, by a restricted set of diagnostic characters—and its success is measured by completeness and by fidelity to a natural order conceived as antecedent to any particular use. This universalist ambition was itself theologically freighted: Linnaeus understood the cataloging of species as the disclosure of a divinely instituted order, and his economy of nature (*Oeconomia Naturae*, 1749) situated classification within a natural theology characteristic of the period (Koerner, 1999). Recent scholarship has also complicated the image of Linnaeus as a purely *a priori* systematist: his practice was intensely empirical—extensive global collecting, some sixteen thousand herbarium specimens, and dedicated information-management techniques (Müller-Wille and Charmantier, 2012; Müller-Wille, 2007) — and the older essentialist reading of his taxonomy has been substantially revised (Winsor, 2003; Larson, 1971). The relevant contrast with Li Shizhen is therefore not simply “Daoist versus Christian” cosmology—both systems are embedded in cosmologies—nor a straightforward opposition of empirical to non-empirical method, but one of pragmatic-empiricist retrieval versus universalist representation.

This distinction reframes several features that might otherwise be read as defects. For example, the inclusion of the dragon among the scaled animals is not best understood as a lapse in empirical rigor. Within a classification ordered by use and by the order of the living world, the dragon occupies a determinate place—as the chief of the scaled creatures and as the source of established materia medica (the fossil drugs *longgu*, “dragon bone,” and *longchi*, “dragon teeth”) — and its inclusion follows from the system’s logic rather than violating it. A universalist taxonomy of organisms must exclude the mythological; a pragmatic-cosmological pharmacopeia has principled reasons to retain it. Recognizing this guards against the anachronism of grading a sixteenth-century materia medica against the membership rules of a system designed three centuries later for a different purpose.

### 3.3 Cosmological ordering and structural analogies with evolutionary thought

Darwin’s theory of evolution by natural selection, articulated in *On the Origin of Species* (1859), holds that biological diversity arises from the gradual modification of ancestral populations over geological time through differential reproductive success (Darwin, 1859). The Compendium predates this theory by three centuries and does not mechanistically anticipate it. Nevertheless, the text exhibits several features that resonate with evolutionary thought in a structural and suggestive sense. It must be emphasized, in response to peer review, that Li Shizhen’s ordering of substances and organisms is grounded in traditional Chinese cosmology—specifically in the graduated ontology of the wǔ-xíng (five phases) and qi frameworks—not in any awareness of biological evolution or common descent. The features discussed below are therefore best understood as cosmological-structural parallels that later readers may find suggestive of evolutionary thinking, not as evidence that Li Shizhen anticipated Darwinian theory. This shared empiricism should not, however, be read as a single axis on which the two systems can be ranked: Linnaeus himself classed his sexual system as artificial, and in both traditions empirical practice operated alongside a non-empirical ordering principle—wǔ-xíng cosmology for Li Shizhen, natural theology for Linnaeus (see Section 3.2.1).

#### 3.3.1 Hierarchical naturalism

The animal kingdom’s sequential order—from insects through fish, reptiles, birds, and beasts to humans—is described by Li Shizhen as proceeding “from humble to noble.” This broadly aligns with what Enlightenment naturalists called the “great chain of being” (scala naturae). It is important to recognize, however, that the scala naturae represents precisely the static, cosmological-hierarchical worldview that Darwinian evolution displaced rather than confirmed. As Mayr (1982) documents, Darwin’s theory of common descent and natural selection replaced the notion of a fixed, vertically ordered chain of being with a branching, genealogical tree in which diversity arises through variation and differential reproductive success—a fundamentally different logical structure with no fixed apex or predetermined direction (Winther, 2008). Li Shizhen’s ordering from “humble to noble” is therefore best understood as reflecting traditional Chinese cosmological thought—in which nature proceeds from the fundamental to the derived—rather than as an anticipation of an evolutionary mechanism. The placement of humans last reflects cultural and philosophical precedent rather than phylogenetic reasoning (Tang, 1989).

#### 3.3.2 Ecological and behavioral observation

The text contains numerous observations on animal behavior, diet, habitat, and ecology. Although originally compiled for pharmacological purposes, this information constitutes a robust body of natural history data that is directly analogous to the ecological observations Darwin drew on when developing his theories. For example, the entries on insects in the Worm Department include careful observations of life cycles, metamorphosis, and habitat preferences that anticipate the kind of comparative biology Darwin later systematized in post-Origin works such as *The Descent of Man* (1871) and *The Expression of the Emotions in Man and Animals* (1872).

#### 3.3.3 Empiricist epistemology

Li Shizhen’s critical methodology embodies an empiricist epistemology that was a necessary precondition for the scientific revolution in both China and Europe. His willingness to reject the authority of earlier texts when they conflicted with his own observations and experiments—including personally tasting herbs—demonstrates a profound commitment to evidence over established dogma. The Compendium*’s* explicit identification and correction of mistakes in earlier *materia medica* parallel the empiricist philosophy underlying both Linnaean taxonomy and Darwinian natural history (Tang, 1989; Unschuld, 1985).

### 3.4 Historical circulation of the compendium in East Asia and Europe

The question of whether the Compendium exerted any direct influence on the development of European taxonomy or evolutionary thought is historically complex. This section traces the routes of textual circulation and should be read as evidence of historical dissemination rather than as evidence of intellectual influence, which cannot be established without direct archival documentation. The text was first transmitted to Japan in 1606, to Korea and Vietnam shortly thereafter, and to Europe in 1656. That year, the Polish Jesuit missionary Michael Boym (1612–1659) translated selections of the text into Latin as *Flora Sinensis*, which was published in Vienna (Needham and Lu, 2000; Boym, 1656). A more extensive Italian translation appeared in Milan in 1676, followed by French, German, English, and Russian versions from 1735 onward.

Active from the 1730s, Linnaeus would have had access to European accounts of Chinese natural history, including Boym’s *Flora Sinensis*. Several scholars have noted that Linnaeus’s *Species Plantarum* (1753) includes descriptions of Chinese plants—some ultimately drawn from the Compendium via intermediate sources—though no direct citation of Li Shizhen’s text appears in Linnaeus’s published works (Linnaeus, 1753). The botanical knowledge encoded in the Compendium thus entered the European taxonomic tradition through a chain of translation and adaptation rather than through direct textual engagement. As Lu (2023) demonstrates in his microhistorical study of the global circulation of Chinese materia medica, such transmission characteristically occurred through networks of intermediary translators, missionaries, and naturalists rather than through direct scholarly citation, making the archival reconstruction of influence both methodologically complex and historically significant.

Darwin’s case is even more equivocal. As noted earlier, the frequently cited claim that he described the Compendium as an “ancient Chinese encyclopedia” (China Medical University, 2026) has not been verified in his published works or surviving correspondence, and the attribution likely stems from a secondary Chinese source. What is clear, however, is that Darwin was broadly aware of the intellectual achievements of Chinese civilization. He drew on a wide range of naturalistic and agricultural traditions—including Chinese horticulture and animal breeding—when developing his arguments for artificial and natural selection in *On the Origin of Species* (1859) and *The Variation of Animals and Plants under Domestication* (1868). Whether the Compendium contributed to this background knowledge, directly or indirectly, remains an open question that warrants further archival investigation.

Regardless of direct causal influence, the parallel development of systematic natural inquiry in sixteenth-century China and eighteenth-century Europe highlights a fundamental feature of scientific knowledge: the hierarchical classification of natural objects appears to be a universal cognitive strategy that emerges independently wherever naturalists seek to make sense of biological diversity. The convergent development of classificatory systems in China and Europe suggests that the Linnaean revolution was not a uniquely Western achievement but rather the crystallization of a universal classificatory imperative that had long been operative across multiple scientific traditions (Stace, 1989; Unschuld, 1985).

## 4 Discussion

### 4.1 The compendium as an encyclopedia: broader contributions to natural science

Beyond its foundational work in taxonomy, the *Bencao Gangmu* makes profound contributions across a range of natural sciences that anticipate later empirical developments. This interdisciplinary scope elevates the text from a purely pharmacological manual to a comprehensive encyclopedia of the natural world.

#### 4.1.1 Mineralogy, zoology, and botany

The text demonstrates remarkable observational rigor across multiple scientific domains. In mineralogy, its sixteen-section treatment of metals and stones details ore formation, crystal habits, and chemical properties; while framed in the vocabulary of Chinese natural philosophy, these insights are firmly grounded in acute empirical observation (Tang, 1989). In zoology, the classification of aquatic animals within the “Scaled” and “Shelled” departments anticipates later systematics by clearly distinguishing between cartilaginous and bony fish, marine and freshwater organisms, and crustaceans and mollusks.

#### 4.1.2 Ecology and phenology

The *Bencao Gangmu* also serves as a significant repository of ecological and phenological science. Its meticulous descriptions of collection seasons—detailing precisely when to harvest roots, bark, leaves, flowers, and fruits to maximize pharmacological potency—serve as applied observations of plant phenology correlated with the agricultural calendar. Furthermore, the text systematically records ecological habitats, noting whether a plant thrives in mountains, valleys, riparian zones, or cultivated gardens, and whether an animal is nocturnal, diurnal, terrestrial, or aquatic. Although assembled for pharmacological purposes, these observations constitute a rich database of ecological knowledge with independent scientific value (Needham and Lu, 2000).

#### 4.1.3 Medical ethics and critical evaluation

The text’s treatment of the human body (Department 52) is ethically complex by modern standards, as it includes historical descriptions of human tissues and waste products used medicinally. However, Li Shizhen expresses explicit reservations about several of these remedies. Noting his scholarly obligation to record historical usages, he cautions that he will “only detail those that are not harmful in principle” while electing to “briefly mention those that are cruel, sinister, or impure” (Li, 1596). This ethical reflection highlights that Li Shizhen was not merely a passive compiler, but a critical evaluator who applied both normative and empirical criteria to his source material.

### 4.2 Educational implications

The *Bencao Gangmu* offers a valuable resource for medical and scientific education in three specific areas: ethnopharmacological history, pharmacognostic methodology, and comparative scientific reasoning. This perspective is supported by recent scholarship on the global transmission of Chinese pharmacological knowledge, which demonstrates that Chinese materia medica circulated across Asia and Europe through sustained networks of translation and adaptation (Lu, 2023; Unschuld, 1985).

#### 4.2.1 Ethnopharmacological history and pharmacognosy

When studying plant-derived drugs such as ephedrine (from *Ephedra sinica* Stapf [Ephedraceae/mahuang]), artemisinin (from *Artemisia annua* L. [Asteraceae/qinghao]), and berberine (from *Coptis chinensis* Franch. [Ranunculaceae/huanglian]), students can trace these compounds directly to their foundational descriptions in the Compendium. This exercise helps learners appreciate a rigorous empirical tradition that systematically documented the morphological, ecological, and therapeutic properties of medicinal substances centuries before modern pharmacology isolated their active principles (Tu, 2016). The text’s standardized eight-section format—recording etymology, geographical distribution, collection seasons, energetic properties, therapeutic indications, and error corrections—offers a concrete illustration of pre-modern pharmacognostic methodology that remains historically instructive.

#### 4.2.2 Comparative scientific methodology

The Compendium’s classification system provides a case study demonstrating that systematic hierarchical natural inquiry emerged independently across distinct cultural and epistemological traditions. Taught alongside standard material on Linnaeus and Darwin, it offers students direct evidence that the classificatory impulse is not a uniquely European achievement, and that empiricist, evidence-based reasoning developed in parallel across the history of science (Needham and Lu, 2000; Unschuld, 1985).

The ongoing digitization of the text through the Chinese Text Project (https://ctext.org) and the Open CourseWare module developed at National Yang Ming Chiao Tung University illustrates one proposed model for integrating this material into university teaching (UNESCO, 2011; Chinese Text Project, 2026). Formal pedagogical assessment of learner outcomes has not yet been conducted; the educational claims advanced here should therefore be understood as evidence-informed proposals rather than demonstrated outcomes, and empirical evaluation is recommended as a next step.

### 4.3 Critical appraisal: limitations and errors in the compendium

A balanced educational engagement with the Compendium must acknowledge its limitations and errors alongside its achievements. The text includes entries on mythological creatures, such as the dragon in the Scaled Department. As argued in Section 3.2.1, such inclusions are better understood as consequences of a classification ordered by therapeutic use and cosmological order than as simple empirical errors: within that framework, the dragon has a determinate place and an associated materia medica, and judging it by the membership rules of a modern organismal taxonomy is anachronistic. The Compendium also includes substances that lack a pharmacological basis by modern standards. Many of its therapeutic claims lack contemporary scientific evidence, and some remedies—particularly those derived from the Human and Beast Departments—are ethically unacceptable in modern practice. Furthermore, Li Shizhen’s cosmological framework, which assigns properties to drugs based on the five phases (wǔ-xíng) theory and the yīn-yáng polarity, is fundamentally incompatible with modern biochemistry and physiology. To place this limitation in historical context: the wǔ-xíng and yīn-yáng frameworks were not mere superstition but sophisticated, internally coherent theoretical systems that structured Chinese natural philosophy for over two millennia. They provided Li Shizhen with a principled vocabulary for categorizing therapeutic properties and ecological relationships that was epistemologically appropriate to his time and tradition. The incompatibility with modern biochemistry reflects a paradigm shift rather than a failure of intellectual rigor; evaluating the Compendium purely by post-eighteenth-century scientific standards risks anachronism in judging a work that must be understood within its own cosmological and medical context (Unschuld, 1985; Needham and Lu, 2000).

Similarly, the comparisons drawn with Linnaeus and Darwin must be contextualized with appropriate caveats. Li Shizhen’s classification lacked a formal nomenclatural code, a strict concept of the type specimen, and a mechanism for resolving conflicts between competing names—features that definitively distinguish modern taxonomy from pre-Linnaean natural history. Additionally, his sequential ordering of organisms from simpler to more complex does not imply a mechanistic theory of evolutionary transformation. Rather, it reflects a traditional Chinese cosmological view of nature as ordered from the fundamental to the derived, and from the simple to the complex, without any necessary implication of chronological or historical sequence (Tang, 1989; Unschuld, 1985). The intellectual connection between the Compendium and modern evolutionary biology is therefore analogical and structural, not causal or genealogical.

Nevertheless, these limitations do not diminish the Compendium’s monumental historical achievement. Every landmark scientific work inevitably contains errors when judged by the standards of subsequent discoveries: Newton’s mechanics required relativistic correction, Linnaeus’s artificial sexual system gave way to natural and phylogenetic classifications, and Darwin’s pangenesis theory was superseded by Mendelian genetics. Therefore, the appropriate standard for evaluating Li Shizhen’s work is the knowledge and methodological framework available in sixteenth-century China. Judged by this metric, the Compendium stands as an extraordinary synthesis of empirical observation, critical evaluation, and systematic organization.

## 5 Conclusion

The *Compendium of Materia Medica* by Li Shizhen (1518–1593) is a significant contribution to the global history of systematic natural inquiry. Its four-tiered classification of 1,892 medicinal substances across three kingdoms—inorganic, plant, and animal—predates the Linnaean taxonomic hierarchy by about 175 years and reflects an empirically grounded, encyclopedic approach to organizing natural knowledge. Although Li Shizhen did not articulate a theory of biological evolution, and although his sequential ordering of organisms reflects traditional Chinese cosmological thought rather than phylogenetic reasoning, the Compendium’s morphological groupings, empiricist critical methodology, and ecologically grounded descriptions of organisms exhibit structural and epistemological features that later readers may find analogous to elements of both Linnaean taxonomy and Darwinian natural history. These parallels are analogical and structural; they do not imply causal connection, theoretical equivalence, or anticipation of Western scientific frameworks.

For medical and scientific education, the Compendium offers a historically grounded case study in ethnopharmacology, pharmacognostic methodology, and the independent emergence of systematic classification across distinct intellectual traditions. Its integration into curricula—through comparative taxonomy exercises, pharmacognosy modules, or history-of-science case studies—can provide students with direct evidence that empiricist, evidence-based reasoning developed across cultures and centuries, rather than within a single tradition.

The intellectual relationship among Li Shizhen, Carolus Linnaeus, and Charles Darwin is not one of direct lineage or theoretical equivalence, but of convergent structural achievement across incommensurable epistemological traditions. That three naturalists—separated by culture, language, and centuries—each independently developed hierarchical, empirically grounded approaches to organizing natural diversity suggests that systematic classification is a broadly human cognitive strategy and that recognizing its global history may enrich the teaching of science.
